# Large-scale functional inference for skin-expressing lncRNAs using expression and sequence information

**DOI:** 10.1172/jci.insight.172956

**Published:** 2023-12-22

**Authors:** Matthew T. Patrick, Sutharzan Sreeskandarajan, Alanna Shefler, Rachael Wasikowski, Mrinal K. Sarkar, Jiahan Chen, Tingting Qin, Allison C. Billi, J. Michelle Kahlenberg, Errol Prens, Alain Hovnanian, Stephan Weidinger, James T. Elder, Chao-Chung Kuo, Johann E. Gudjonsson, Lam C. Tsoi

**Affiliations:** 1 Department of Dermatology, Michigan Medicine, University of Michigan, Ann Arbor, Michigan, USA.; 2 Center for Autoimmune Genomics and Etiology (CAGE), Cincinnati Children’s Hospital Medical Center, Cincinnati, Ohio, USA.; 3 College of Sciences, North Carolina State University, Raleigh, North Carolina, USA.; 4 Department of Computational Medicine & Bioinformatics and; 5 Division of Rheumatology, Department of Internal Medicine, University of Michigan, Ann Arbor, Michigan, USA.; 6 Department of Dermatology, Erasmus University Medical Center, Rotterdam, Netherlands.; 7 Laboratory of Genetic Skin Diseases, Imagine Institute, Paris, France.; 8 Department of Dermatology and Allergy, University Medical Center Schleswig-Holstein, Kiel, Germany.; 9 Ann Arbor Veterans Affairs Hospital, Ann Arbor, Michigan, USA.; 10 Institute for Computational Genomics, Joint Research Center for Computational Biomedicine, RWTH Aachen University, Aachen, Germany.; 11 Department of Biostatistics, University of Michigan, Ann Arbor, Michigan, USA.

**Keywords:** Dermatology, Genetics, Bioinformatics, Genetic variation, Skin

## Abstract

Long noncoding RNAs (lncRNAs) regulate the expression of protein-coding genes and have been shown to play important roles in inflammatory skin diseases. However, we still have limited understanding of the functional impact of lncRNAs in skin, partly due to their tissue specificity and lower expression levels compared with protein-coding genes. We compiled a comprehensive list of 18,517 lncRNAs from different sources and studied their expression profiles in 834 RNA-Seq samples from multiple inflammatory skin conditions and cytokine-stimulated keratinocytes. Applying a balanced random forest to predict involvement in biological functions, we achieved a median AUROC of 0.79 in 10-fold cross-validation, identifying significant DNA binding domains (DBDs) for 39 lncRNAs. *G18244*, a skin-expressing lncRNA predicted for IL-4/IL-13 signaling in keratinocytes, was highly correlated in expression with *F13A1*, a protein-coding gene involved in macrophage regulation, and we further identified a significant DBD in *F13A1* for *G18244*. Reflecting clinical implications, *AC090198.1* (predicted for IL-17 pathway) and *AC005332.6* (predicted for IFN-γ pathway) had significant negative correlation with the SCORAD metric for atopic dermatitis. We also utilized single-cell RNA and spatial sequencing data to validate cell type specificity. Our research demonstrates lncRNAs have important immunological roles and can help prioritize their impact on inflammatory skin diseases.

## Introduction

Less than 2% of the human genome is translated into proteins (1); however, 8%–15% is estimated to be functional (2, 3), and results from ENCODE suggest as much as 80% of the genome can have biological activity (4). Advances in high-resolution sequencing technologies have enabled deep molecular profiling, identifying elements that were not previously revealed due to their cell type specificity and low-expressing nature. Long noncoding RNA (lncRNA; 200 or more nucleotides) is arguably the gene category that has expanded the most in the genomic era, from 9,640 in 2012 (5) to 19,928 in 2022 (6). lncRNAs are of particular interest (7) for their ability to regulate gene expression (8) and alter splicing control (9) by binding to target DNA; there is also some evidence that lncRNAs may act as a scaffold to recruit proteins (10, 11). Therefore, lncRNAs have been shown to affect the regulation of different biological mechanisms; however, few lncRNAs have been functionally characterized (12), and much remains to be discovered regarding their role in health and disease.

Specific lncRNAs have been found to operate in different skin conditions (13), such as *ANRIL* for melanoma (14), *HOTAIR* for systemic sclerosis (15), *TINCR* for burn injuries (16) and epidermal differentiation more generally (17), *MALAT1* for squamous cell carcinoma (18), and *WAKMAR1* for wound healing (19). Our previous study (20) revealed that many skin-expressing lncRNAs — including lncRNAs *G2608* and *G25746* — are dysregulated in lesional skin of patients with psoriasis. Intriguingly, compared with previously annotated lncRNAs, a greater proportion of the 1,080 potentially novel skin-expressing lncRNAs we identified were differentially expressed in psoriasis. lncRNAs are highly tissue specific (21), and this can be beneficial for precision medicine. For example, drug-response models trained using lncRNAs outperformed protein-coding models for several medications (22). However, tissue specificity — along with unique spatiotemporal dynamics (23), lower overall expression, and poor sequence conservation (24) (compared with protein-coding transcripts) — can make the biological roles of lncRNAs difficult to determine.

Various approaches have been applied to predict lncRNA biological functions. Proximity to coding genes (e.g, direct sense-antisense pairing; refs. 25, 26) can reveal the function of some lncRNAs. Sequence motifs have also been used to associate with subcellular localization or protein binding (27). For example, *XIST* contains a repeat E motif, without which it is unable to interact with CIZ1 for enrichment in the nucleus (28). However, this approach requires careful analysis to understand how specific motifs may influence the function of each lncRNA. Automated techniques are increasingly being developed that apply machine learning to gene coexpression (29) or generic sequence features such as k-mer profiles (30, 31). It is also possible to predict lncRNA disease associations through matrix completion of similarity based on disease-gene and disease-ontology pairs (32). However, all these approaches depend on the quality and quantity of existing data, which is particularly limited for skin conditions and cutaneous biology.

In our study, we apply machine-learning techniques to a large collection of RNA-Seq data sets from different in vivo and in vitro experiments to predict lncRNAs for specific cytokine pathways; we then utilize the sequence information to refine the regulatory effects. Our study provides a comprehensive catalog for lncRNA genomics and highlights the potential roles of lncRNAs in inflammatory skin diseases.

## Results

### lncRNA are differentially expressed in inflammatory skin diseases.

**Figure 1:**
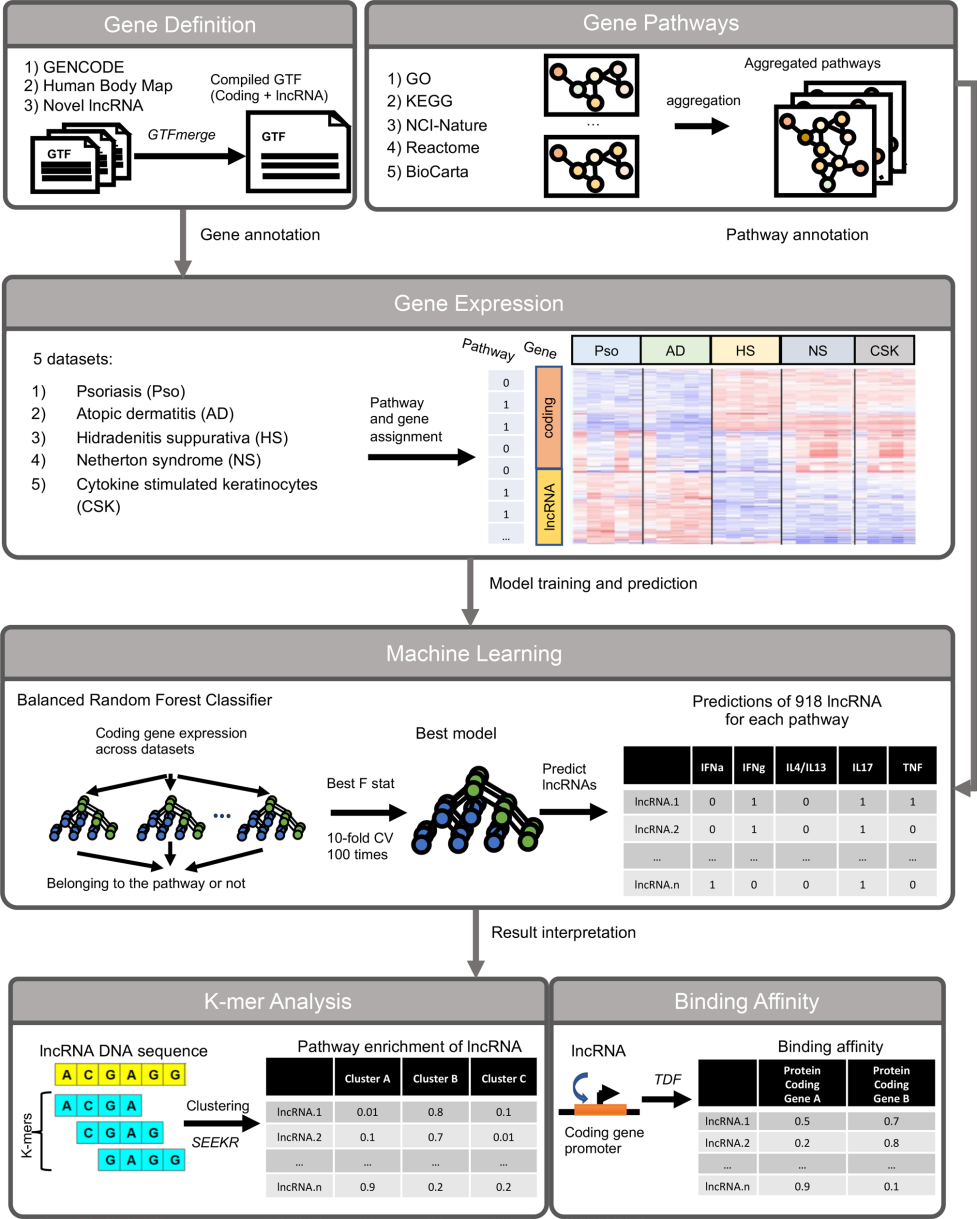
Overview of our approach. Workflow describing the different data sources and steps in our prediction and analysis of lncRNA functions.

Figure 1 provides an overview of the workflow for our approach. We profiled gene expression in 434 transcriptomes from 4 inflammatory skin disease RNA-Seq cohorts (atopic dermatitis [AD], psoriasis, Netherton syndrome, and hidradenitis suppurativa [HS]), revealing 11,082 lncRNAs and 18,022 protein-coding genes to be expressed in at least 1 skin condition (Table 1). Of these, 12,547 genes — including 4,163 (33%) lncRNAs — were significantly (FDR ≤ 0.05) differentially expressed in at least 1 condition (Supplemental Tables 1–4; supplemental material available online with this article; https://doi.org/10.1172/jci.insight.172956DS1). Interestingly, for all 4 diseases, a higher proportion of the skin-expressing lncRNAs was differentially expressed compared with protein-coding genes, suggesting that cellular and molecular changes at the tissue level might have a higher regulatory effect on genes with high cell type specificity, and their overall expressions can be affected by cellular composition changes (33, 34). Importantly, we identified a core set of 51 lncRNAs that were dysregulated in the same direction (14 upregulated and 37 downregulated) for all 4 skin diseases (Supplemental Figures 2 and 3).

Gene expression was further evaluated in cytokine-stimulated (IFN-α, IFN-γ, IL-4, IL-13, IL-17, IL-17 + TNF, and TNF) keratinocytes and unstimulated keratinocytes (50 samples of each condition). Of the 4,543 lncRNA and 15,406 protein-coding genes expressed (Supplemental Table 5), 2,179 genes — including 429 (20%) lncRNAs — were significantly (FDR ≤ 0.05) differentially expressed in at least 1 stimulation (Supplemental Tables 6–12). By contrast with the skin conditions, the proportions of skin-expressing lncRNAs that are differentially expressed were consistently lower than those for protein-coding genes for all 7 cytokine stimulations. For each pair of cytokines and skin conditions, we counted the number of shared differentially expressed lncRNA (Supplemental Table 13) and found a total of 17 pairs to be significantly enriched after correcting for multiple testing through Bonferroni adjustment, including IFN-γ in psoriasis (*P* = 6.1 × 10^–18^, OR = 3.37) and IL-13 (*P* = 1.1 × 10^–5^, OR = 63.90) in AD.

Of the 4,163 lncRNAs (5%) differentially expressed in a skin condition, 217 were differentially expressed in a cytokine simulation, including 5 of the 51 lncRNAs (10%) that have the same direction of effect in all 4 skin conditions. Interestingly, these 5 lncRNAs were more likely to be upregulated rather than downregulated in the skin conditions (*P* = 0.018, OR = 13.1, Fisher enrichment test). We also calculated the distance of each of the 51 lncRNAs to H3K27ac marks for active enhancers (35) and the nearest protein-coding gene expressed in a cytokine stimulation (Supplemental Table 14); however, no significant differences were found comparing lncRNAs dysregulated/not dysregulated in at least 1 cytokine condition.

### Predicting cytokine pathways in skin for lncRNAs.

To predict the potential functions of the lncRNAs, we compiled multiple gene sets (Supplemental Table 15) relevant to pathways of cytokine response from 5 different sources: BioCarta (36), Reactome (37), NCI (38), GO (39), and KEGG (40). Aggregating these gene sets provides 135 protein-coding genes for the IFN-α pathway, 239 for IFN-γ, 120 for IL-17, 195 for IL-4/IL-13, and 330 for TNF. We selected these genes as the positive set to predict the involvement of lncRNAs for each cytokine signaling pathway, using the aforementioned RNA-Seq samples from inflammatory skin diseases and cytokine stimulations as training data. An additional 129 samples, available from the same data sets (taken, for example, from nonlesional sites), were included such that the training data had 834 samples in total.

**Figure 2:**
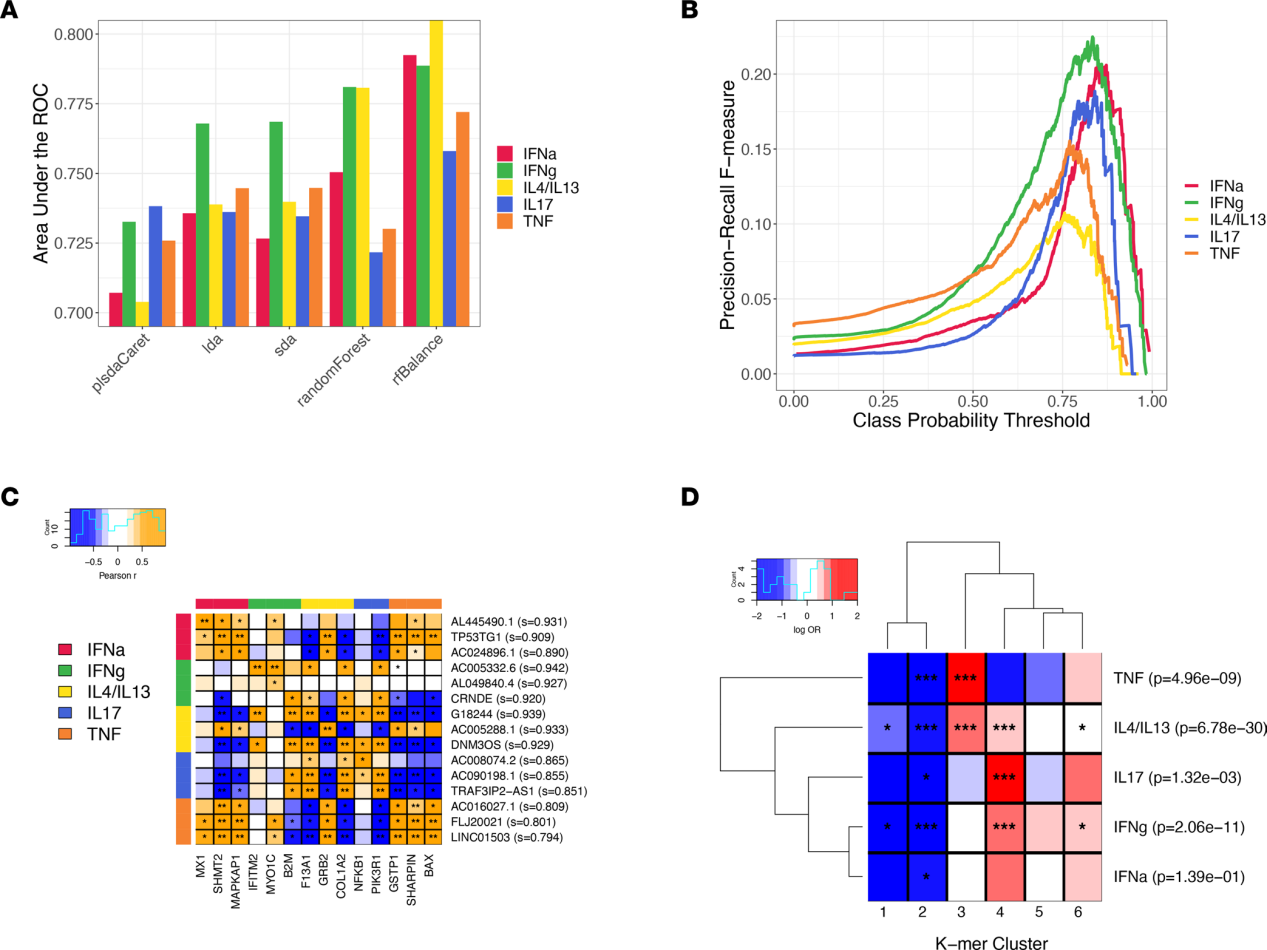
Results of lncRNA function prediction. (**A**) Benchmarking the performance of classifiers across different cytokine pathways on the prediction task, and measuring according to the AUROC on protein-coding genes. (**B**) The F-measure curve for protein-coding genes in each cytokine pathway, as a harmonic mean of precision and recall, is used to set the threshold for lncRNA prediction. (**C**) Heatmap of correlations between the top 3 lncRNA for each cytokine pathway and the most correlated protein-coding genes. *P < 1e-10; **P ≤ 1e-100. (**D**) Enrichment of lncRNA predicted for different cytokines in k-mer clusters from SEEKR analysis. *nominally significant (*P* < 0.05); ***significant after Bonferroni (p_adj < 0.05).

Machine-learning prediction was conducted using 10-fold cross-validation to help reduce the risk of overfitting. We benchmarked 10 different classifiers on the cytokine pathways using the protein-coding genes (Figure 2A and Supplemental Figure 4) and found they gave consistently satisfactory performance, with 8 of 10 classifiers having an area under the receiver operating characteristic (AUROC) greater than 0.6 for all 5 pathways. The highest-performing classifier overall was random forest, which had a median AUROC (across the pathways) of 0.75. We further found that, when setting the number of cases and controls in each tree of the random forest to be equal (i.e., balanced), this significantly increased the median AUROC to 0.79 in cross-validation (paired Wilcoxon *P* = 0.031). When dividing the data into 70% training and 30% testing, the balanced random forest achieved 0.80 AUROC for IFN-α, 0.79 for IFN-γ, 0.79 for IL-4/IL-13, 0.71 for IL-17, and 0.74 for TNF.

Applying the balanced random forest classifier to predict which lncRNAs participate in each cytokine pathway, we averaged the prediction scores for every gene over 100 trials (Supplemental Table 16). *AL445490.1*, the lncRNA with the highest prediction score for IFN-α (mean score = 0.931), is antisense and positioned intronic and upstream of IFN-α–inducible protein 6 (*IFI6*) and was previously found to be highly expressed in an inflammatory subtype of breast cancer (41). Indeed, we found the expression of *AL445490.1* to be highly correlated with *IFI6* (Spearman *r* = 0.79, *P* = 1.5 × 10^–175^) across our keratinocyte and skin disease samples. The top predicted lncRNA for other cytokines are differentially expressed and/or play roles in different kinds of cancer: *AC016027.1* for TNF (mean score = 0.81 of 1) in colorectal cancer (42, 43); *AC005332.6* for IFN-γ (mean score = 0.94) in pancreatic cancer (44); and *AC008074.2* for IL-17 (mean score = 0.87) in bladder cancer (45). Meanwhile, the highest prediction for the IL-4/IL-13 pathway, *G18244* (mean score = 0.94), was identified as a skin-expressing lncRNA in our previous study (20). Interestingly, single-cell RNA-Seq (scRNA-Seq) analysis on 17 skin samples (46) demonstrates that *AC016027.1* and *AC008074.2* are more expressed in keratinocytes, while *G18244* has greater expression in endothelial cells (Supplemental Figure 5–7).

We set a prediction score threshold for each cytokine pathway by maximizing the F-statistic (Figure 2B). The F-statistic represents the harmonic mean of precision (the proportion of genes predicted to be involved in a pathway that actually are involved) and recall (the proportion of genes involved in the pathway that are successfully predicted to be involved), thus providing an optimal trade-off between these 2 competing goals. We calculated the maximum F-statistic for each cytokine pathway using the protein-coding genes and set the threshold 1 percentage point lower (to account for statistical error); we then included all lncRNAs with mean prediction scores greater than this in the final set of lncRNAs predicted for each cytokine (Supplemental Table 17). Table 2 presents a summary of these results and shows that we have predicted 27 lncRNAs for IFN-α, 148 for IFN-γ, 683 for IL-4/IL-13, 25 for IL-17, and 35 for TNF, with 165 lncRNAs being predicted for more than 1 pathway and 721 lncRNAs overall (Supplemental Figure 8). We compared the predicted lncRNAs with those differentially expressed for each cytokine (Supplemental Table 18) and found significant enrichment (after Bonferroni correction) among lncRNAs predicted for IFN-α (*P* = 5.5 × 10^–8^, OR = 750.8), IFN-γ (*P* = 3.80 × 10^–21^, OR = 14.2), and IL-17 (*P* = 4.0 × 10^–3^, OR = 383.5).

Exploring a possible further refinement, we aggregated interaction scores (from the RNAInter v4.0 database) between lncRNAs and the coding genes in each pathway; we then correlated these with the prediction scores from our machine-learning framework. Interestingly, the correlation for all 5 cytokine pathways — IFN-α (*r* = 0.26, *P* = 3.2 × 10^–182^), IFN-γ (*r* = 0.24, *P* = 2.5 × 10^–153^), IL-17 (*r* = 0.33, *P* = 3.7 × 10^–308^), IL-4/IL-13 (*r* = 0.33, *P* = 5.0 × 10^–296^), and TNF (*r* = 0.27, *P* = 5.4 × 10^–191^) — was strong, although it is difficult to evaluate the effect on predictions without a gold standard for lncRNA functions.

### Assessing the roles of predicted lncRNAs.

Figure 2C presents the top 3 lncRNAs predicted for each cytokine pathway, along with the protein-coding genes that have the highest correlation in gene expression with each one, across the different samples in the training data set. For the IL-17 pathway, 2 of the top 3 lncRNAs, *AC090198*.*1* and *TRA3IP2-AS1*, have the highest correlation with the protein-coding gene *PIK3R1*, so there are only 2 protein-coding genes shown. As expected, lncRNAs tend to have a higher correlation with protein-coding genes in their predicted pathways (Figure 2C), and when extending to all predicted lncRNAs, we found they had consistently and significantly (after Bonferroni correction) higher mean correlation with protein-coding genes connected with the cytokines they are predicted for compared with those from other cytokines (Supplemental Table 19). Interestingly, in the IL-4/IL-13 pathway, *AC005288.1* has a strong positive correlation with *GRB2* and a weaker negative correlation with the other 2 protein-coding genes (*F13A1* and *COL1A2*), while for lncRNAs *G18244* and *DNM3OS*, it is the opposite. IL-4 and IL-13 antagonize IFN-γ and TNF via *GATA3* and *STAT6* (47); accordingly, we found *AC005288.1* to be negatively correlated with *GATA3* and *STAT6*, while *G18244* and *DNM3OS* were positively correlated. There is also some overlap in the correlations between the different cytokine pathways, with lncRNAs predicted for IFN-α also being correlated with protein-coding genes involved in TNF and vice versa. To evaluate the clinical implication of our analysis, we studied whether the top-prediction results would be biomarkers for disease severity. Interestingly, we observed multiple instances where the expressions of lncRNAs in AD lesional skin are significantly correlated with SCORAD, a clinical tool used to assess extent and severity (48), including *AC090198.1* (*P* = –0.48; *P* = 1 × 10^–4^) and AC005332.6 (*P* = –0.42; *P* = 1 × 10^–4^).

**Figure 3:**
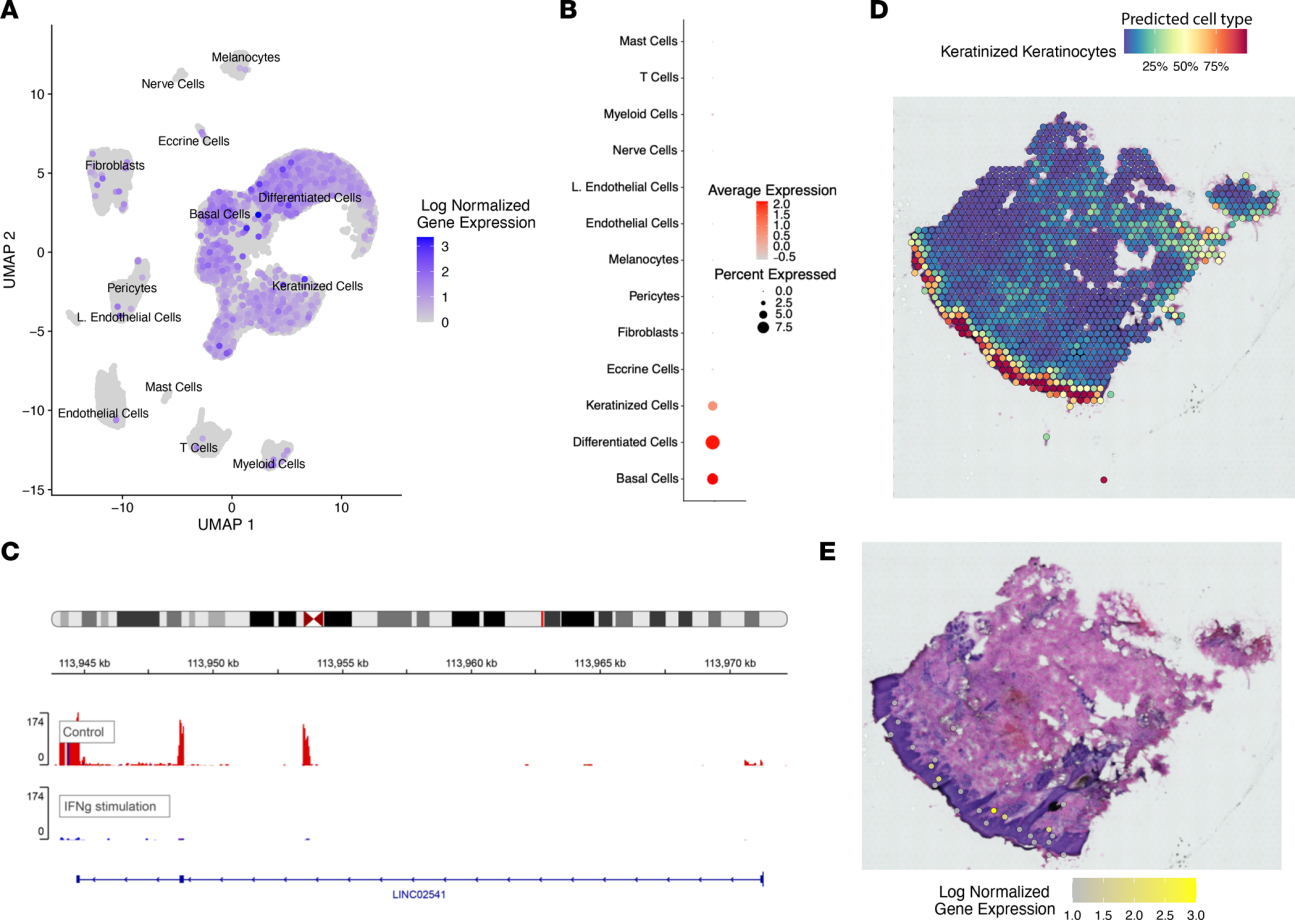
Investigation of *LINC02541*. (**A**) UMAP plot of scRNA-Seq analysis, with color scale representing normalized gene expression in each cell. (**B**) Dot plot of scRNA-Seq analysis, with color scale representing average expression and size representing percentage of cells in which the gene is expressed. (**C**) Coverage plot comparing read counts for a keratinocyte sample stimulated with IFN-γ against control, illustrating downregulation upon cytokine stimulation. (**D**) Spatial plot of keratinized keratinocyte predicted proportions shows the approximate location of keratinocytes within the tissue sample. (**E**) Histology image, confirming *LINC02541* (indicated in yellow) is expressed mostly by the keratinocytes.

Another lncRNA of interest, *LINC02541*, predicted by our machine-learning approach to be involved in the IFN-γ pathway (mean score = 0.822), was significantly negatively correlated (*r* = –0.628, *P* = 9.5 × 10^–93^) with *STAT3*, a gene known to be involved in the pathogenesis of psoriasis (49), among other inflammatory skin diseases. Using scRNA-Seq, Figure 3, A and B, we show that *LINC02541* is specifically expressed in keratinocytes, and we found it to be significantly downregulated (*P* = 1.6 × 10^–20^, FC = 0.48) in keratinocytes upon stimulation with IFN-γ (Figure 3C), while *STAT3* was significantly upregulated (*P* = 2.1 × 10^–38^, FC = 3.31). We used spatial transcriptomic data to further assay the expression profile of *LINC02541* in skin, highlighting that its expression is mostly in the basal/differentiated compartments in epidermis (Figure 3, D and E). Furthermore, *LINC02541* was consistently downregulated in psoriasis (*P* = 2.6 × 10^–20^, FC = 0.54), AD (*P* = 4.0 × 10^–10^, FC = 0.54), and Netherton syndrome (*P* = 1.0 × 10^–3^, FC = 0.55), while *STAT3* was upregulated in all 3 diseases. These results suggest a potential regulatory role in skin inflammation for *LINC02541* through STAT3-mediated IFN-γ signaling and demonstrate the utility of our approach to reveal previously unknown roles of lncRNAs.

**Figure 4:**
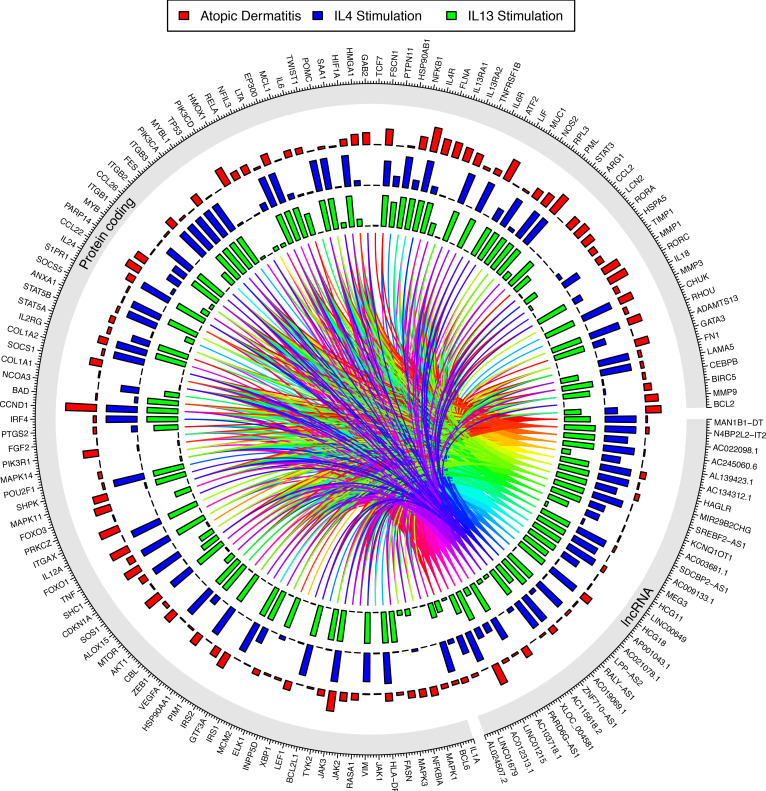
Circos plot showing RGT-TDF predictions for IL-4/IL-13. The outer circle indicates the different lncRNA predicted by our machine-learning approach and the protein-coding genes annotated as being involved in the pathway. Inside are bar plots showing the –log_10_ *P* values from the differential expression for atopic dermatitis (red), IL-4 stimulation (blue), and IL-13 stimulation (green). The innermost part of the plot shows the links between the lncRNAs and the protein-coding genes for the predicted DNA binding domains (DBDs), color-coded to distinguish the different lncRNAs.

The highest correlation we observed was between one of our skin-expressed lncRNAs, *G18244*, and the protein-coding gene *F13A1* (*r* = 0.978, *P* < 5 × 10^–324^) in the IL-4/IL-13 pathway. *F13A1* encodes subunit A of factor XIII, which plays important roles in skin pathophysiology (50). *F13A1* is involved in macrophage regulation (51, 52), which can be stimulated by IL-4 (53, 54). To further investigate the link between *G18244* and *F13A1*, we applied the Triplex Domain Finder (TDF) from the Regulatory Genomics Toolbox (RGT) (55), which tests for DNA binding domains (DBDs) between lncRNAs and DNA promoter regions. Applying this approach to *G18244*, we identified a significant DBD (OR = 171.8, *P* = 4 × 10^–2^) that encompasses the promoter for *F13A1*. Significantly, when screening putative DBD for *G18244* against promoters for 500 randomly selected protein-coding genes, there were no significant DBDs. We expanded our RGT-TDF analysis to the lncRNAs predicted for each pathway (under the F-statistic thresholds), using the protein-coding genes from each respective cytokine pathway for the target promoters, and we found significant DBDs for 38 lncRNAs predicted for the IL-4/IL-13 cytokine pathway, 2 for IFN-γ, and 1 for TNF, with 39 unique lncRNAs in total (Supplemental Table 20). Five of the lncRNAs predicted for IL-4/IL-13 (*AC022098.1*, *AL139423.1*, *KCNQ1OT1*, *LINC00861*, and *MEG3*) had multiple significant DBDs (Figure 4).

Inspired by previous work (30), which suggests that short motif sequences of lncRNAs (k-mers) may provide clues for their function, we counted the number of different 6-mers for each lncRNA in our combined catalog. Then, we performed community detection using SEEKR (30), an efficient tool that performs hierarchical clustering based on the Pearson correlation metric. SEEKR identified 6 distinct clusters of lncRNAs (Figure 2D), one of which (cluster 2) is enriched among lncRNAs predicted for all 5 cytokine pathways. By contrast, cluster 3 appears to be specific to TNF and IL-4/IL-13, while cluster 4 was more enriched among lncRNAs predicted to be involved in IL-17 and IFN-γ. This supports previous research claims that sequence motifs in lncRNAs can affect their function, confirms the distinctiveness of lncRNAs predicted by our approach, and suggests some grouping among lncRNAs involved in different cytokine pathways.

## Discussion

Previous work has demonstrated that lncRNAs can play important roles in inflammatory skin diseases (20); however, little is known regarding the specific immune functions of these genes. By combining multiple gene catalogs with our own skin-expressing transcripts from the largest available data set (>200 RNA-Seq lesional and normal skin samples), we increased the number of lncRNAs to investigate by 30%. Other sources of potentially novel lncRNA annotations are available; however, many of these overlap with our data set (e.g., >99% for LNCipedia; ref. 56); we decided to focus primarily on established and skin-expressing lncRNAs in this study; we also acknowledge that newer versions of GENCODE are available since the time of our analysis. Interestingly, we found that a higher proportion of skin-expressing lncRNAs was significantly differentially expressed for skin diseases compared with protein-coding genes, whereas, for cytokine stimulations in keratinocytes, the converse was true. This simultaneously highlights the importance of lncRNAs for skin diseases and the difficulties in interpreting their function. lncRNAs are especially difficult to analyze because they tend to have lower expression levels than protein-coding genes, and their expression patterns are highly context specific. We countered these difficulties by preparing a large data set of RNA-Seq gene expression (834 samples in total) across 4 skin diseases and 7 cytokine stimulations; we then applied advanced machine-learning techniques to predict lncRNA functions, using promoter binding analysis and sequence motifs (k-mers) to interpret the results.

We applied a machine-learning approach to predict lncRNAs involved in pathways of response to IFN-α, IFN-γ, IL-4/IL-13, IL-17 and TNF, using protein coding genes from these pathways as training data, since lncRNAs’ expression has been found to correlate positively with their coding targets (57–59). The highest-performing classifier in our benchmarking analysis across the different cytokines was random forest, and we further improved its performance (from 0.75 to 0.79 AUROC) by requiring all the trees in the classifier to be balanced — i.e., having an equal number of cases and controls. This helps account for bias in the proportion of cases and controls, with far fewer protein-coding genes being annotated as involved in a cytokine pathway than not. In addition to splitting the data 70/30 for training and testing, we confirmed the robustness of our approach and addressed the potential risk of overfitting by using 10-fold cross-validation, repeating the classification analysis 100 times to obtain an average prediction score for each lncRNA in each cytokine pathway. We also observed consistently high performance for the different classifiers, with 8 of 10 having AUROC > 0.6 for all 5 pathways. Nevertheless, there may be measurement errors due to the methodology employed, and the outcome cannot be considered a ground truth.

Since RNA-Seq samples from cytokine stimulations were used in the training data, we tried excluding them to test whether they could give an unfair advantage to predictions for cytokine pathways (Supplemental Table 21); however, the new prediction scores were highly correlated (*r* > 0.8 for every cytokine) with those that include these samples, suggesting that inclusion of the cytokine stimulations did not overly bias the results. Furthermore, we trained the balanced random forest classifier using 200-dimension gene embeddings created from 984 data sets in GEO (60) and compared the prediction results with our own for the 1,166 lncRNAs available in that data set. Although the Pearson correlations were significant for IFN-γ, IL-4/IL-13, and TNF (but not IFN-α or IL-17), they were much smaller in size (*r* < 0.3 for every cytokine) and smaller also than those for the protein-coding genes (which were all significant), highlighting the tissue specificity of lncRNAs and the utility of our own large skin/keratinocyte RNA-Seq data set.

The lack of previous research into lncRNAs in skin diseases can be a limitation on interpreting the findings of our study. Most previous studies have focused on specific lncRNAs (15, 19), so when researching the highest predicted lncRNAs for each cytokine, literature was limited to reports of putative roles in cancer. These previous studies confirm that lncRNAs can have an immune role, but their involvement in skin biology is a potentially novel finding. Some of the lncRNAs we identified (e.g., *G18244*) are new to our knowledge, but we can understand their mechanisms of action through correlation with protein-coding genes (for example, *F13A1*) and provide further confirmation through promoter testing using RGT-TDF analysis. In addition, k-mer clustering and correlation analysis was used to help reveal patterns and provide insight into the role of sequence motifs. Overall, our study provides a large body of functional predictions and research findings, which can be used to guide future research into the roles of lncRNAs in inflammatory skin diseases.

## Methods

### Merged gene catalog.

We compiled a comprehensive gene catalog, including 18,517 lncRNAs, by combining data from GENCODE v29 (6) with Broad’s Human Body Map (21) and the skin-expressing transcripts identified in our previous study (20). This increased the number of lncRNAs by 31% compared with using GENCODE alone (Supplemental Figure 1), bringing it closer to the 20,110 protein-coding genes. Our gene catalog also includes 14,881 pseudogenes; 7,517 short noncoding RNAs; and 1,424 other genes, which we group together into the “other” category.

Gene transfer format (GTF) files were merged across the 3 data sets using our custom software, GTFmerge (https://github.com/CutaneousBioinf/GTF; commit ID 44a323b), which is written in C++ for efficiency. GTFmerge only adds genes that are nonoverlapping, by checking whether any of the exons from the target GTF overlap with an exon from the reference GTF. First, we set GENCODE as the reference and the Human Body Map as the target; then, we set the merged GTF as the reference and our skin-expressing transcripts as the target, so as to create a new merged GTF comprising all 3 sources.

### RNA-Seq data processing.

RNA-Seq samples were processed, as described in their respective papers, for AD (61), psoriasis (20), Netherton syndrome (62), and HS (34), using STAR for alignment and HTSeq to count the number of reads for each gene in our merged gene catalog. In addition to the lesional/control samples used in differential expression analysis (Table 1), additional samples were included in machine learning: 6 acute AD, 27 nonlesional AD, 28 psoriasis lesional, and 27 psoriasis nonlesional from ref. 61; 28 nonlesional psoriasis from ref. 20; and 13 nonlesional Netherton from ref. 62. Differential expression analysis was performed using limma (63). We compared lesional skin with control samples from healthy patients and included all available covariates (sex for AD; sex and age for Netherton syndrome and HS). Genes were considered differentially expressed if they have |logFC| ≥ 1 and FDR ≤ 0.05. Single-cell and spatial data were extracted from a previous study (64) and analysis performed to reveal the cell and spatial specificity of particular lncRNAs. Spatial deconvolution of cell types was performed using conditional autoregressive-based deconvolution (65).

### Machine learning.

We performed supervised machine learning for functional prediction, using 10-fold cross-validation on a range of different classifiers (rpart, naiveBayes, logreg, ksvm, ctree, gbm, plsdaCaret, lda, sda, and randomForest) through the MLR package in R, using default parameters for each. We performed 5 binary classification tasks, 1 for each cytokine pathway (aggregated from gene sets; Supplemental Table 15), with genes as the instances and disease/cytokine stimulation expression profiles (samples) as the features. Each task was trained using the expression of all genes on all samples, so the size of the training set was the same for each task: 20,110 protein-coding genes, with 834 samples. For the balanced random forest, we sampled all genes involved in the pathway and an equal number not involved. Protein-coding genes annotated as belonging to each cytokine in the aggregated pathways were marked as positive cases, and the other protein-coding genes were marked as negative controls for the purposes of classification; hence, our approach can be considered a form of positive-unlabeled (PU) classification. Expression data were inverse normalized for use in machine learning. The resulting models were then applied to predict lncRNA for each cytokine pathway, using the maximum F-statistic to set thresholds on the prediction score for each cytokine.

### Promoter testing and k-mer clustering.

We applied the TDF from the RGT (55) using the hg19 reference genome and the default input parameters (promoter length: 1,000; minimum length of triplex: 20; triplex-forming promoter cut-off: 0.1; tolerated number of consecutive errors: 2). The k-mer clustering tool SEEKR (30) was applied using k-mers of length 6 and the default threshold for the adjacency matrix (0.13). We then calculated enrichments of lncRNAs predicted for different cytokine pathways using Fisher’s exact test.

### Statistics.

We performed various statistical tests, including Spearman/Pearson correlation, Fisher enrichment, and Wilcoxon rank-sum tests. Where appropriate, Bonferroni adjustment was used to correct for multiple tests.

### Study approval.

Since our study focused on the analysis and utilization of previously collected data, no prior approval was required.

### Data availability.

The GENCODE v29 GTF can be downloaded from the GENCODE website (https://www.gencodegenes.org/human/release_29.html). The additional lncRNA GTF has been deposited in GEO (GSE63979), as has the AD (GSE224783), psoriasis (GSE63979), Netherton syndrome (GSE164285), and HS (GSE154773) expression data. Values for all data points in graphs are reported in the Supporting Data Values file.

## Author contributions

Design of research studies was contributed by MTP and LCT. Data were acquired by MKS, JEG, and LCT. Data were analyzed by MTP, SS, AS, and RW. The original draft was written by MTP and LCT. Review and editing were contributed by MTP, SS, AS, RW, MKS, JC, TQ, ACB, JMK, EP, AH, SW, JTE, CCK, JEG, and LCT.

## Supplementary Material

Supplemental data

Supplemental tables 1-18

Supporting data values

## Figures and Tables

**Table 1:**
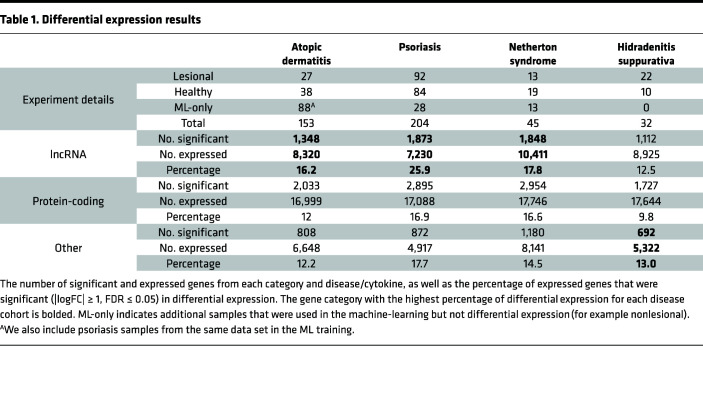
Differential expression results

**Table 2:**
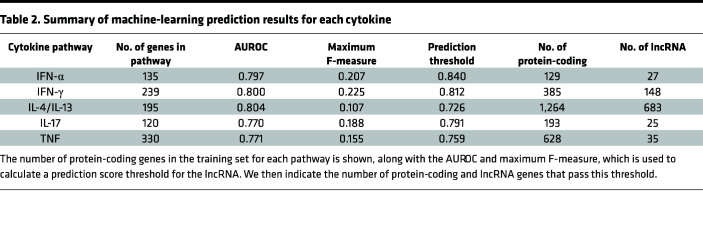
Summary of machine-learning prediction results for each cytokine

## Version 1 12/22/2023

Electronic publication
