# Supplementary material for: Large-scale functional inference for skin-expressing lncRNAs using expression and sequence information
Source: JCI Insight. 2023 Dec 22;8(24):e172956. doi: 10.1172/jci.insight.172956 (PMC10807743; doi:10.1172/jci.insight.172956)
Supplement: Supplemental data [file jciinsight-8-172956-s162.pdf]

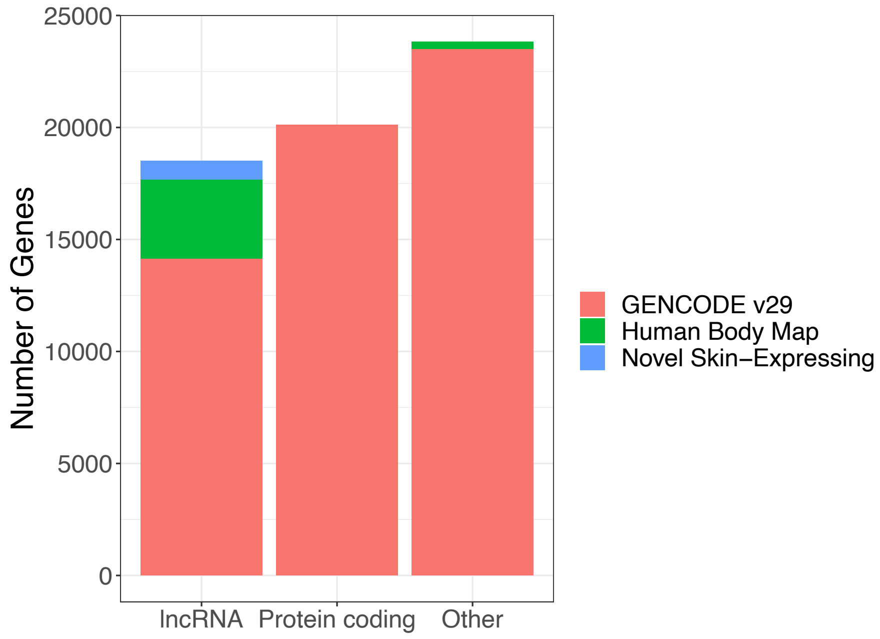

Supplementary Figure 1: Histogram of skin-expressed gene types. We increased the number of lncRNA in our combined gene catalog by 30% compared to using GENCODE alone.

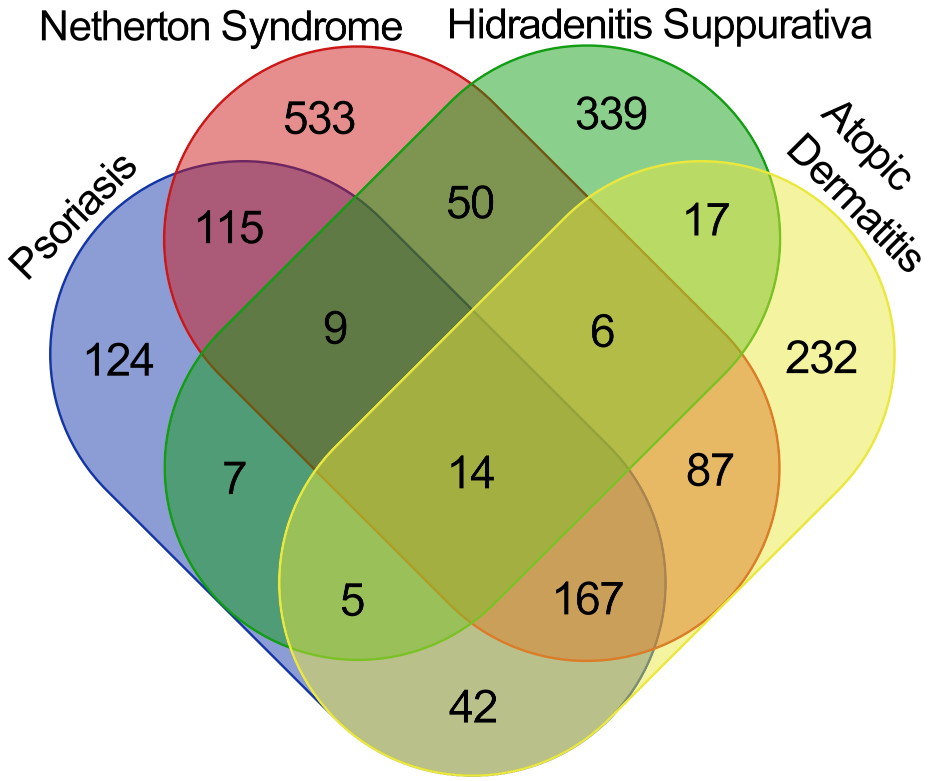

Supplementary Figure 2: Up-regulated lncRNA shared by each disease. The following 14 lncRNA are shared by all diseases: *AC004585.1*, *AC008063.1*, *AC018716.2*, *AL355312.3*, *CERNA2*, *G13883*, *G25251*, *G31721*, *G39634*, *MIR31HG*, *TRG-AS1*, *XLOC_003595*, *XLOC_004197*, *XLOC_004198*.

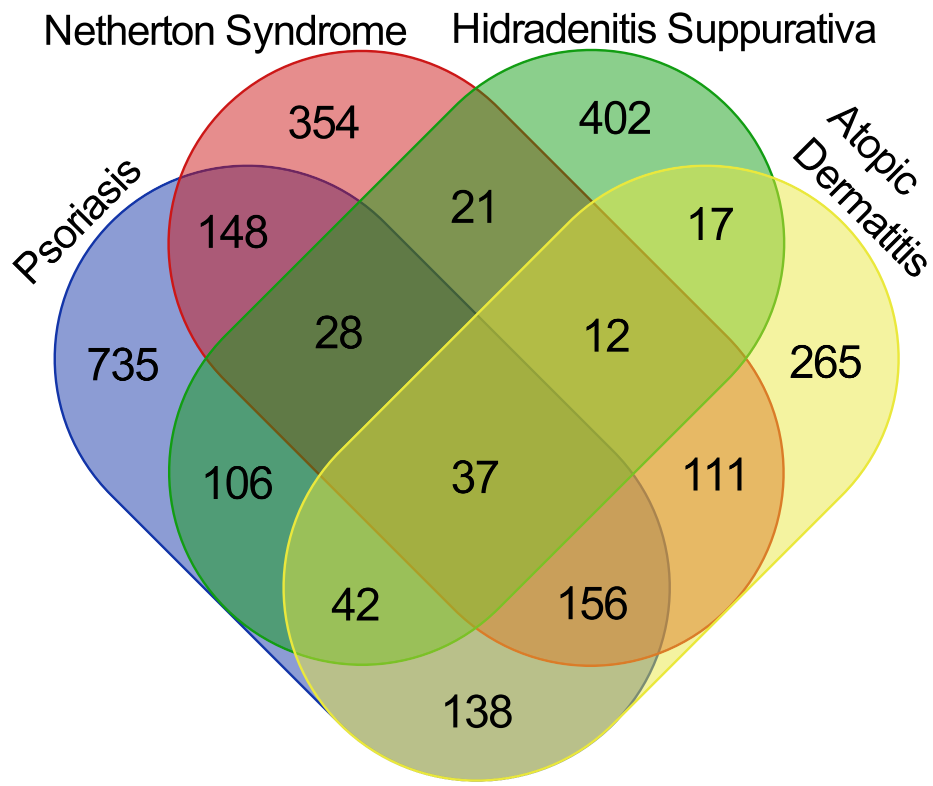

Supplementary Figure 3: Down-regulated lncRNA shared by each disease. The following 37 lncRNA are shared by all diseases: *AC022784.1*, *AC027763.2*, *AC064807.2*, *AC087242.1*, *AC091074.1*, *AC092279.1*, *AC092542.1*, *AC092691.1*, *AC092802.1*, *AC092802.3*, *AC103957.2*, *AC109462.2*, *AC112206.2*, *AC124312.2*, *AC124312.5*, *CNTFR-AS1*, *G13446*, *G14609*, *G21034*, *G2523*, *G30952*, *G32202*, *G33836*, *G33837*, *G34070*, *G37465*, *G43312*, *G4403*, *G6793*, *G9514*, *LINC01266*, *LINC02169*, *XLOC_003775*, *XLOC_004299*, *XLOC_004545*, *XLOC_005173*, *XLOC_011942*.

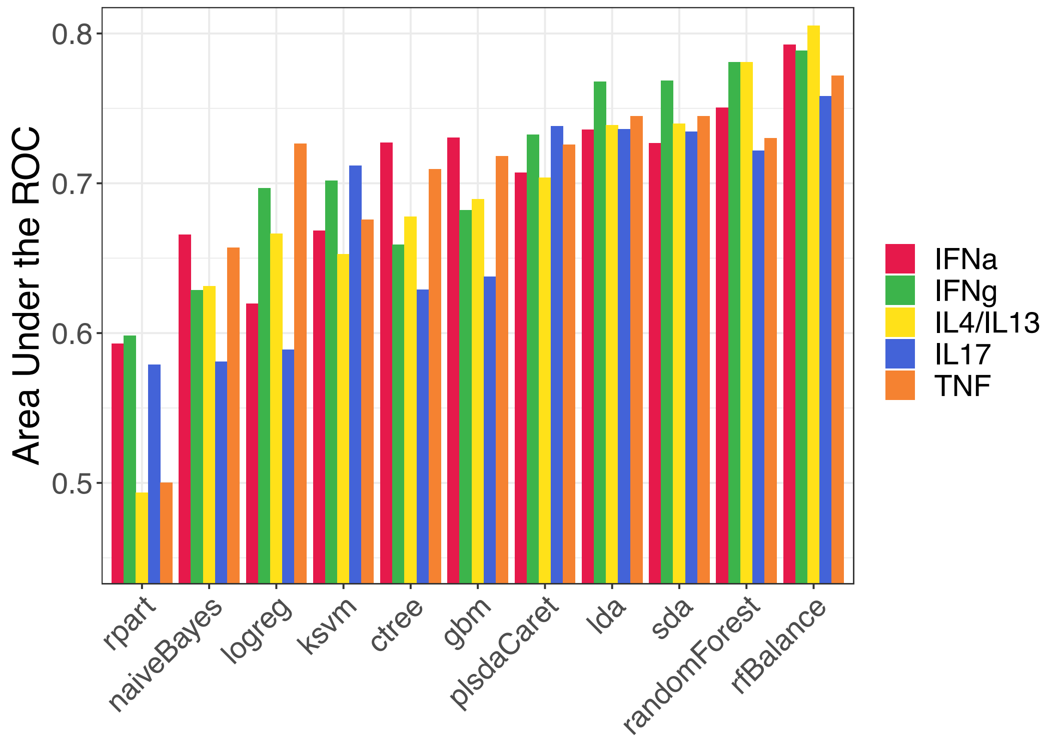

Supplementary Figure 4: Benchmarking the performance of different classifiers across different cytokine pathways on the prediction task. Measuring according to the area under the ROC (AUROC) on protein-coding genes.

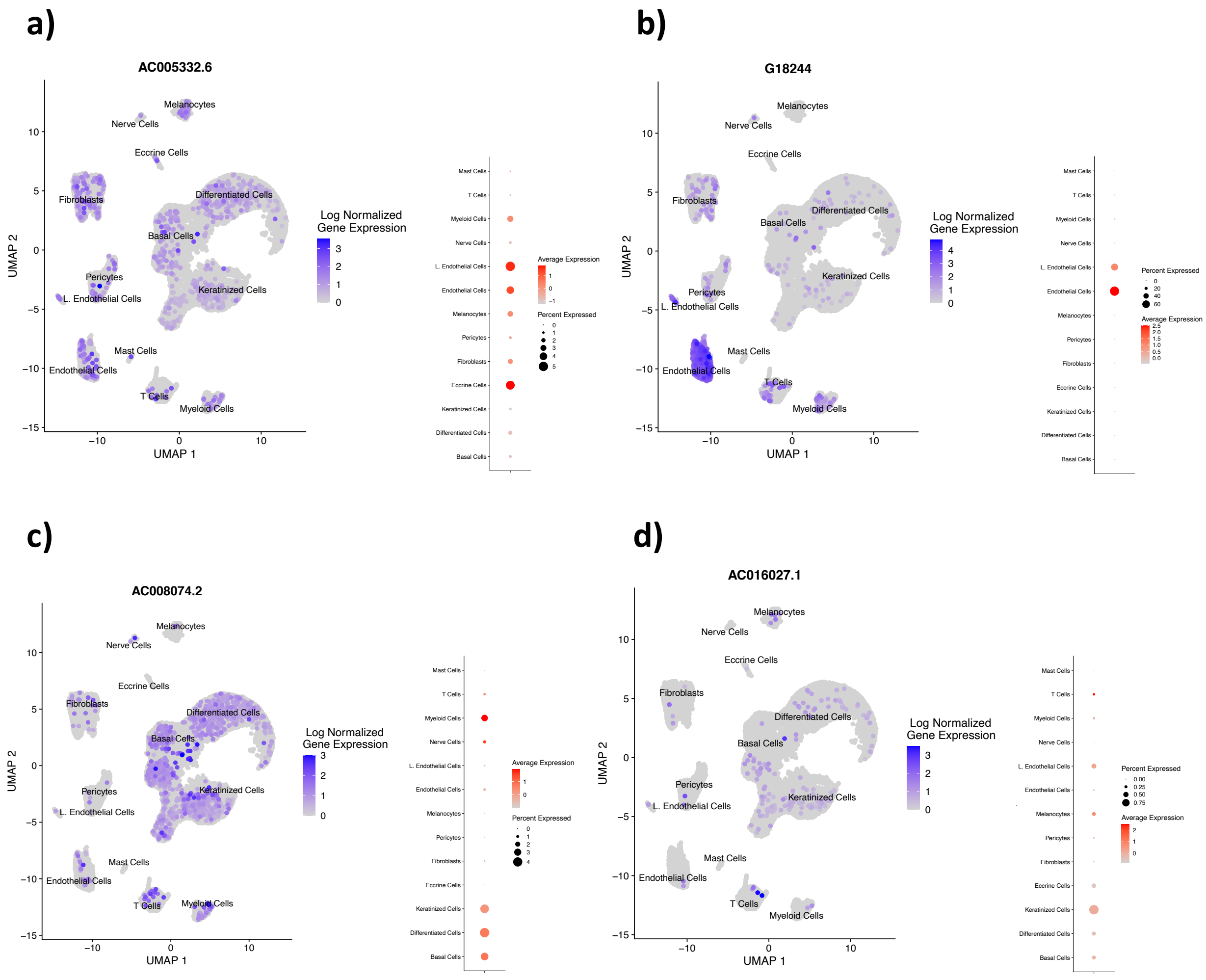

Supplementary Figure 5: UMAP and dot plot of scRNA-seq analysis of the highest predicted lncRNA for each cytokine. The UMAP color scale represents normalized gene expression in each cell, the dot plot color scale represents average expression and dot plot size represents percentage of cells in which the gene is expressed, of: a) *AC005332.6*, the top lncRNA for IFNg; b) *G18244*, the top lncRNA for IL4/IL13; c) *AC008074.2*, the top lncRNA for IL17; and d) *AC016027.1*, the top lncRNA for TNF. *AL445490.1*, the top lncRNA for IFNa, is not shown as it was not expressed in scRNA-seq.

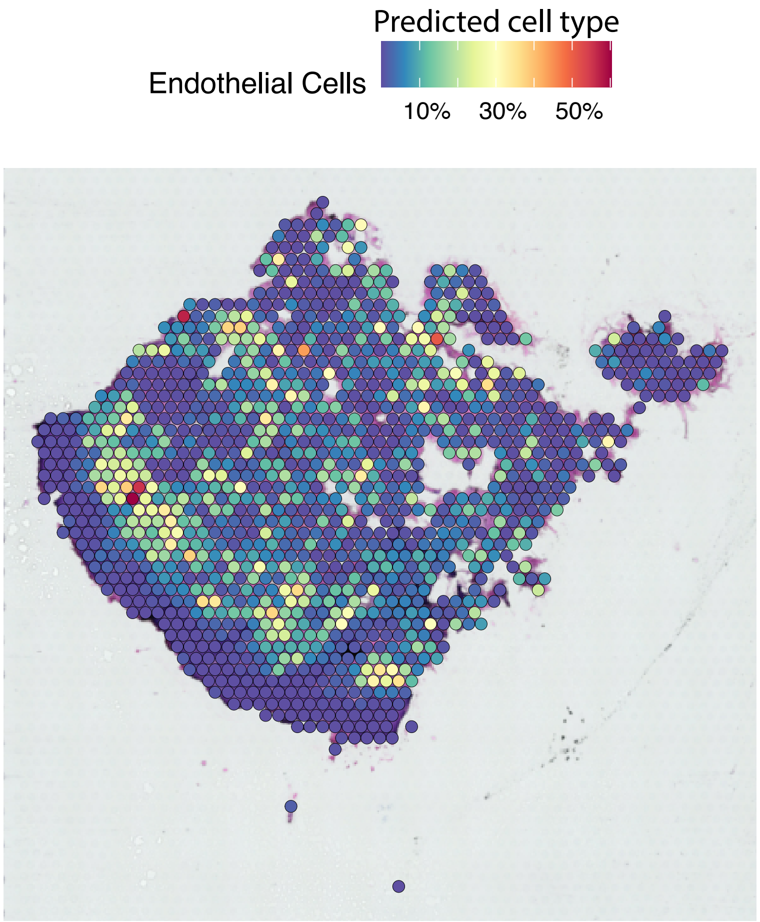

Supplementary Figure 6: Spatial plot of endothelial cell predicted proportions. Shows the approximate location of endothelial cells within the tissue sample.

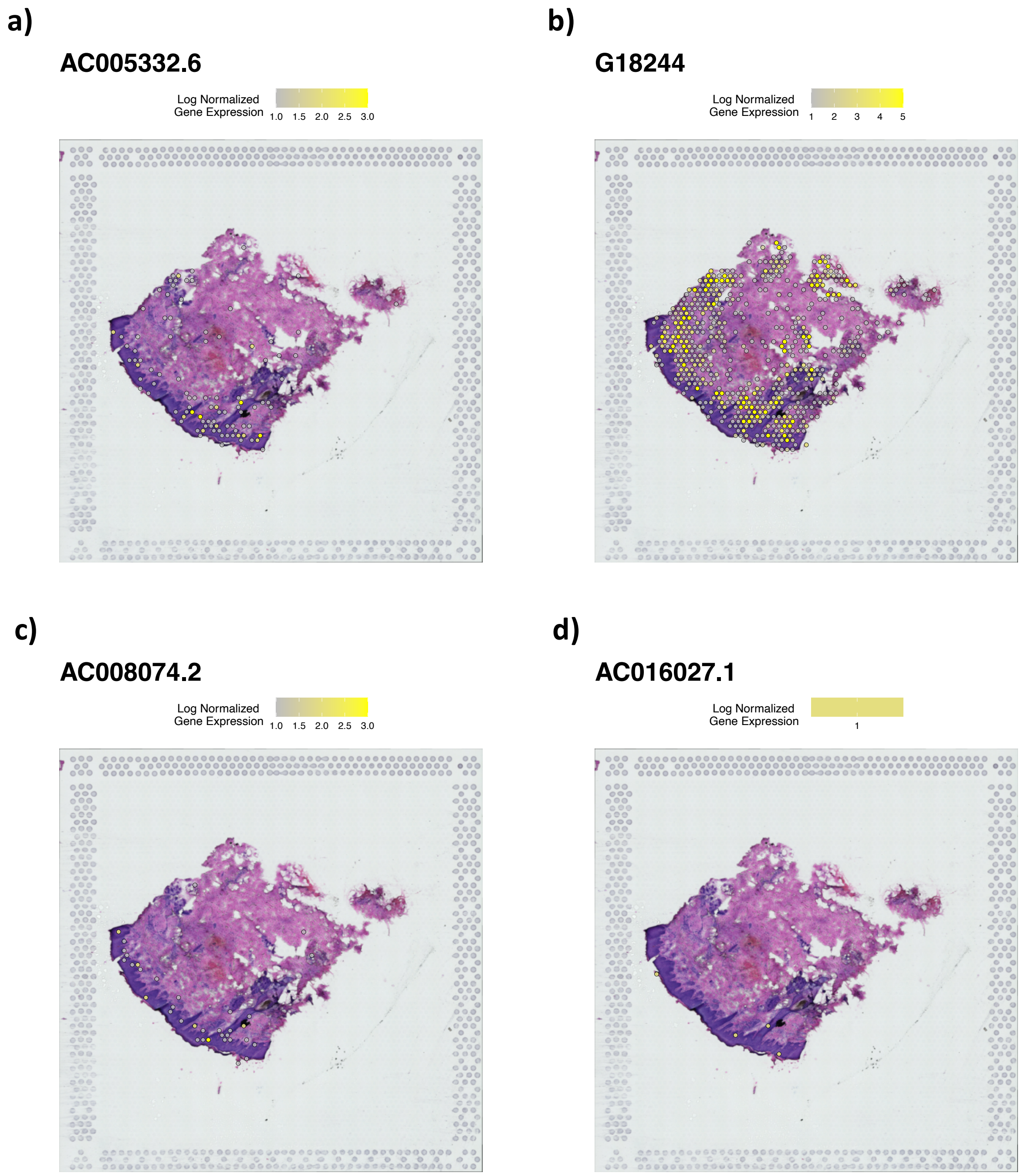

Supplementary Figure 7: Histology image annotated separately with expression of different lncRNA. *AC005332.6*, *AC008074.2* and *AC016027.1* are confirmed to be expressed mostly in keratinocytes, while *G18244* is expressed mostly in endothelial cells.

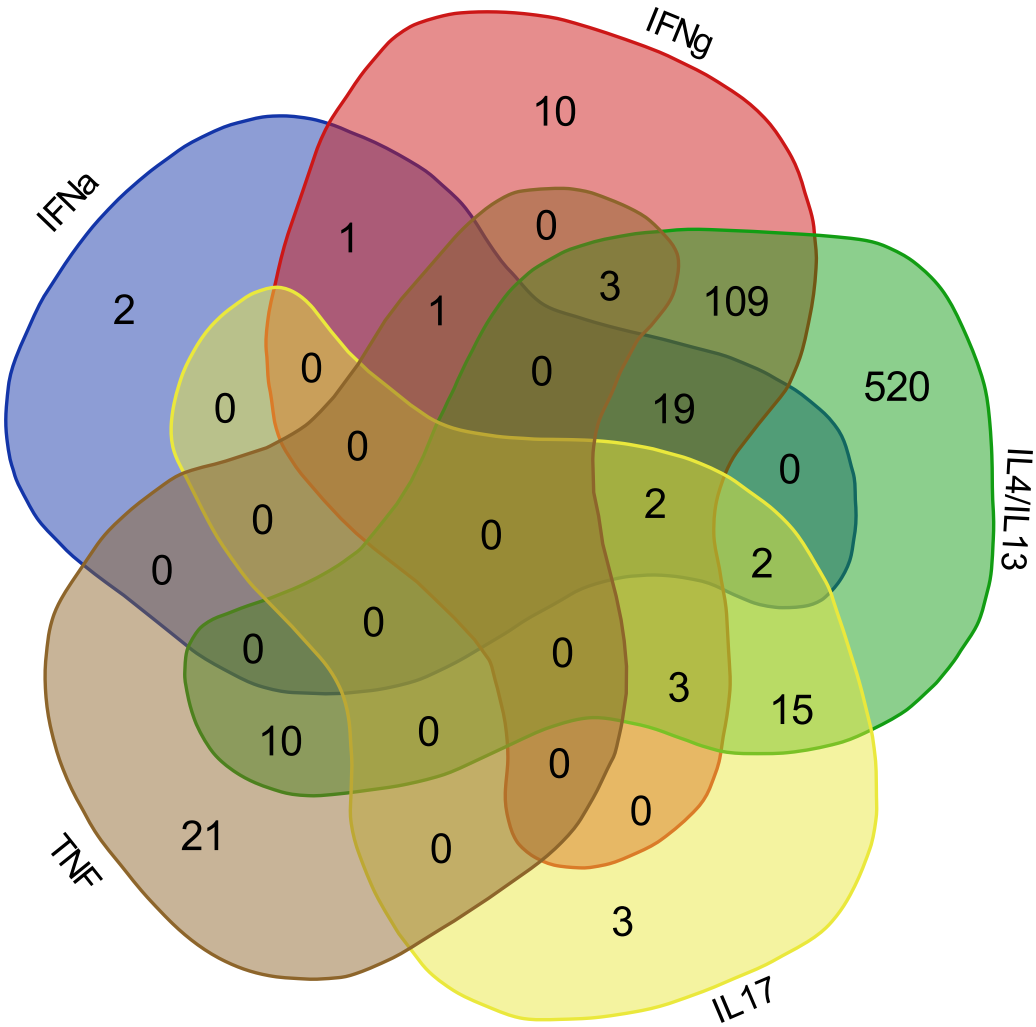

Supplementary Figure 8: Venn diagram showing the overlapping lncRNA predicted for each cytokine. The lncRNA predicted for each cytokine are shown shaded in a different color, with the number of lncRNA provided for each overlap between cytokines.
